# The mitochondrial genome of an ectoparasitoid wasp, *Habrobracon hebetor* (Hymenoptera: Braconidae: Braconinae)

**DOI:** 10.1080/23802359.2020.1787268

**Published:** 2020-07-14

**Authors:** Hao Su, Baoqian Lyu, Rui Meng, Hui Lu, Jihong Tang

**Affiliations:** a College of Plant Protection, Hainan University, Haikou, China; b China Academy of Tropical Agriculture Sciences/Key Laboratory of Integrated Pest Management on Tropical Crops, Ministry of Agriculture and Rural Affairs, Environment and Plant Protection Institute, Haikou, China; c Post-Entry Quarantine Station for Tropical Plant, Haikou, PR China; d Hainan Province Engineering Research Center for Quarantine, Prevention and Control of Exotic Pests, Haikou, PR China

**Keywords:** Braconini, mitogenome, gene rearrangement, phylogenetic relationship

## Abstract

In this study, we sequenced the mitogenome of *Habrobracon hebetor*, and obtained almost complete mitogenome of it. The mitogenome contains 14,629 bp which consists of 13 protein-coding genes (PCGs), 20 transfer RNA genes (*trnI* and *trnM* are absent), and 2 ribosomal RNA genes (GenBank accession no. MT558946). Gene rearrangement events occurred in this species, five tRNA genes with changes in positions or/and directions are found. All of 13 PCGs started with ATN. Eight PCGs used the typical stop codon ‘TAA’, five PCGs terminated with incomplete stop codons (T). Phylogenetic analyses within the Cyclostomes were performed based on mitochondrial PCGs.

*Habrobracon hebetor* (Say) (Hymenoptera: Braconidae: Braconinae: Braconini) is a cosmopolitan gregarious, idiobiont, larval ectoparasitoid of pyralid and noctuid moths (Magro and Parra 2001; Abedi et al. 2012). It is considered one of the most important biological control agents of pyralid moths in stored products. To date, no mitogenome had been studied for the tribe, and no complete mitogenome of Braconinae reported.

In this study, adult samples of *H. hebetor* were obtained in the insectarium of Environment and Plant Protection Institute, China Academy of Tropical Agriculture Sciences, Hainan, China (110°20′9″N, 19°59′21″E). The specimens were deposited at −20 °C in the herbarium of Post-Entry Quarantine Station for Tropical Plant, Haikou Customs District, PR China (speciemen accesion number IN07070201-0000-0010). A single individual was used for DNA extraction.

The mitogenome sequence of *H. hebetor* only obtained 14,629 bp (GenBank accession number MT558946), contained 13 protein-coding genes (PCGs), 20 tRNA genes, and 2 rRNA genes. Two tRNA (*trnI* and *trnM*) and A + T rich region were absent. The nucleotide composition of the known mitogenome sequence was biased toward AT 84.8%, and the base composition was 42% A, 42.8% T, 6.8% G, and 8.4% C.

Gene rearrangement events occurred in this species. All rearranged genes were tRNA. The tRNA arrangement patterns of *trnW*–*trnY*–*trnC* and *trnD*–*trnH*(–)–*trnK* in the sequenced region were found. These rearrangement patterns of tRNAs were consistent with previous results (Dowton 1999; Wei et al. 2010; Zhang et al. 2020).

The length of 13 PCGs was 10,877 bp, all PCGs started with ATN, ATA for *nad2*, *cox1*, *cox2*, *atp8*, *cox3*, *nad3*, and *nad5*; ATG for *atp6*, *nad4*, *nad6*, and *cob*; ATT for *nad4l* and *nad1*. Five PCGs (*atp6*, *nad3*, *nad5*, *nad4*, and *cytb*) terminated with incomplete stop codons (T), the rest PCGs used the typical stop codon ‘TAA’. All of the 20 tRNAs have the usual clover-leaf secondary structure, except for *trnS1*. All tRNAs had normal lengths, which varied from 64 to 71 bp. The 16S rRNA was 1291 bp long with an AT content of 88.5%, while the 12S rRNA only sequenced 365 bp long.

We performed phylogenetic analyses of *H. hebetor* with 10 other species representing 10 subfamilies of Cyclostomes and one species from Helconinae as outgroup based on only 12 PCGs on account of most known mitogenomes of Cyclostomes lost the gene ND2 (Figure 1). The analyses were performed with Bayesian inference MrBayes 3.2.3 (Ronquist et al. 2012) and maximum likelihood in RAxML 8.2.10 (Stamatakis 2014). The results of phylogenetic analysis within Cyclostomes were similar to the previous study (Li et al. 2016). However, as the only two mitochondrial genomes of Braconinae reported so far, *H. hebetor* and *Euurobracon breviterebrae* did not form a monophyletic lineage in the phylogenetic tree, and the result showed they were close to Opiinae and Gnamptodontinae. These results may indicate that more mt-genome sequences are required to resolve the phylogenic relationships within Cyclostomes in further studies.

**Figure 1.**
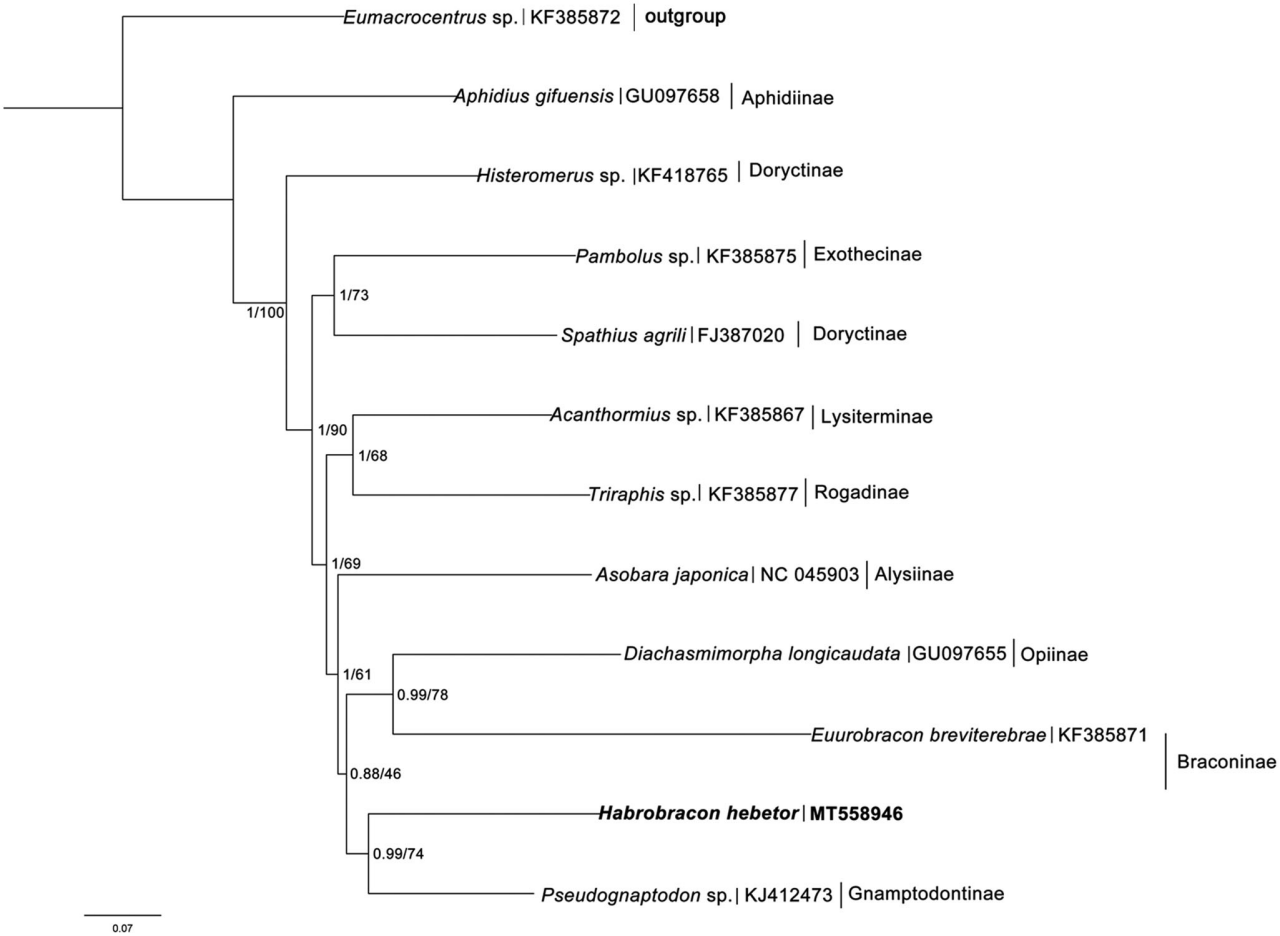
Phylogenetic relationships within Cyclostomes inferred from nucleotide sequences of mitochondrial protein-coding genes, using Bayesian/ML methods.

## Data Availability

The data that support the findings of this study are openly available in NCBI at https://www.ncbi.nlm.nih.gov/, reference number [MT558946], or available from the corresponding author.
